# Expression of *SDF-1* and *CXCR4* transcript variants and *CXCR7* in epithelial ovarian cancer

**DOI:** 10.3892/ol.2014.1897

**Published:** 2014-02-20

**Authors:** KAROLINA JASZCZYNSKA-NOWINKA, MARCIN RUCINSKI, AGNIESZKA ZIOLKOWSKA, ANNA MARKOWSKA, LUDWIK K. MALENDOWICZ

**Affiliations:** 1 Department of Oncology, Poznań University of Medical Sciences, Poznań 60-781, Poland; 2 Department of Histology and Embryology, Poznań University of Medical Sciences, Poznań 60-781, Poland; 3 Department of Perinatology and Gynecology, Poznań University of Medical Sciences, Poznań 60-781, Poland

**Keywords:** *SDF-1*, *CXCR4*, *CXCR7*, transcript variants, epithelial ovarian cancer, plasma SDF-1α, plasma CA 125

## Abstract

Chemokine stromal cell-derived factor-1 (SDF-1) and its receptors, CXCR4 and CXCR7, have been implicated in epithelial ovarian cancer progression and metastasis. However, limited data are available on the expression levels of *SDF-1* and *CXCR4* variants and *CXCR7* in human epithelial ovarian cancer. The present study aimed to characterize the expression pattern and levels of *SDF-1*, *CXCR4* and *CXCR7* in normal human ovaries and epithelial ovarian cancer. The expression of *SDF-1* and *CXCR4* transcript variants and CXCR7 was determined by quantitative polymerase chain reaction (qPCR). Plasma SDF-1α levels were determined by commercially available EIA kits and cancer antigen 125 (CA 125) levels were quantified by automated microparticle enzyme immunosorbent assay. High expression levels of *SDF-1* transcript variant 1 were identified in ovarian cancer and control ovaries. By contrast, in both groups the expression levels of *SDF-1* transcript variants 3 and 4 were extremely low. Furthermore, *SDF-1* variant 1 levels were notably higher in epithelial ovarian cancer than in control ovaries, while data for the remaining transcripts were similar in both groups. *CXCR4* transcript variant 2 and *CXCR7* expression levels in normal and neoplastic ovaries were similar. In both groups, *CXCR4* transcript variant 2 was not detected. Plasma SDF-1α levels were notably higher in females with epithelial ovarian cancer than in the control ovaries. Elevated levels of blood SDF-1α were found prior to surgery, 6 days after surgery and following completion of the first chemotherapy course. These increases were independent of the type of epithelial ovarian cancer. Our results suggest that the expression of *SDF-1* and the genes controlling alternative splicing are elevated in epithelial ovarian cancer, leading to an increased formation of *SDF-1* variant 1. Elevated plasma SDF-1α levels in epithelial ovarian cancer patients are not associated with the presence of tumors and/or metastases, however reflect a general response to the disease.

## Introduction

Stromal cell-derived factor-1 (SDF-1 or CXCL12) is a cytokine belonging to the CXC chemokine family (1,2). For a number of years it was thought that CXCR4 was the sole receptor for this chemokine. However, in 2005, a new SDF-1 receptor was identified and named CXCR7 (3). The SDF-1 and CXCR4/CXCR7 axis exerts pleiotropic activity and is involved in normal development, organogenesis, regeneration and tumorigenesis (4–6).

The chemokine SDF-1 and its receptors, CXCR4 and CXCR7, have been implicated in cancer progression and metastasis. At the mRNA and protein levels, enhanced expression of *SDF-1* and *CXCR4* has been demonstrated in different malignancies, including epithelial ovarian cancer and ovarian cancer cell lines (7–12). These earlier observations on the chemokine expression profile in ovarian cancer were confirmed more recently by other groups (13,14). With regard to the expression of the SDF-1 and CXCR4/CXCR7 axis in ovarian cancer, the majority of data originates from immunohistochemical studies. However, commonly used antibodies are only able to recognize subpopulations of studied molecules (15–18).

Numerous human genes are known to have transcript variants and among them *SDF-1* (Gene ID: 6387) has four variants; *CXCR4* (Gene ID: 7852) has two variants and *CXCR7* (Gene ID: 57007) has no known alternative splicing variants. Limited data are available on the expression levels of *SDF-1* and *CXCR4* variants and *CXCR7* in human epithelial ovarian cancer. Therefore, the present study aimed to characterize the expression pattern and levels of *SDF-1* and *CXCR4* transcript variants and *CXCR7* in epithelial ovarian cancer and healthy human ovaries. We also aimed to correlate the obtained data to plasma SDF-1α levels and to specific clinicopathological variables of studied patients.

## Materials and methods

This study included 113 females subjected to surgery at the Department of Oncology, Poznań University of Medical Sciences (PUMS; Poznań, Poland). Histopathological examinations were conducted at the Department of Tumor Pathology, Wielkopolska Centre for Oncology (Poznań, Poland). The studies were conducted as accepted by the Bioethical Commission (PUMS; consent no.164/11 of 17th February, 2011). Written informed consent was obtained from all patients.

### Study groups and material sampling

#### Expression of SDF-1 and CXCR4 transcript variants and CXCR7 in epithelial ovarian cancer and control ovaries

This study was conducted in 27 patients subjected to surgery in the Department of Oncology (PUMS) due to ovarian cancer. The mean age of the patients in this group was 62 years (range, 47–85 years). The most frequent histopathological diagnosis in the group involved serous carcinoma (18 patients), followed by endometriod carcinoma (four patients), mucinous carcinoma (three patients) and macrocellular carcinoma (two patients). Cancers at stage III of clinical advancement, according to the International Federation of Gynecology and Obstetrics (FIGO), were exhibited in 18 patients, followed by cancers at stage II (four patients), stage IV (three patients) and stage I (two patients). The most frequent were cancers of a low degree of differentiation (grade 3, 13 patients; grade 2, 11 patients; grade 1, three patients). The control group consisted of 13 females subjected to surgery due to uterine myomas or uterine prolapse after menopause. The mean age of the control group patients was 63 years (range, 51–77 years).

During surgery, a section of ovarian tumor or normal ovary tissue was taken (~0.5 cm^3^). The fragments were immersed in RNAlater fluid (Life Technologies, Austin, TX, USA) and frozen at a temperature of −70°C.

#### Plasma concentrations of SDF-1α and cancer antigen 125 (CA 125)

This study was conducted in 43 patients subjected to surgery at the Department of Oncology (PUMS). In this group, the mean age of the patients was 57 years (range, 41–84 years). The most frequent histopathological diagnosis involved serous carcinoma (29 patients), followed by mucinous carcinoma (five patients), endometriod carcinoma (three patients), solid carcinoma (two patients), macrocellular carcinoma (one patient) and other tumors (three patients). The majority of patients were diagnosed with stage III cancer according to FIGO, followed by patients with stage I (6 patients), stage II (2 patients) and stage IV (2 patients). Poorly differentiated cancers consttuted the majority exhibited by the patients (grade 3, 27 patients; grade 2, 13 patients; grade 1, three patients). The control group consisted of 30 females subjected to surgery at the Department of Oncology (PUMS) for gynecological reasons distinct from ovarian cancer and with no neoplastic diseases in anamnesis. The mean age of the control patients was 45 years (range, 31–72 years). In patients with ovarian cancer, venous blood was sampled from the antecubital vein on three occasions: One day prior to surgery, on the 6th day following surgery and after six cycles of chemotherapy. In the control group, blood was sampled once, prior to surgery. The blood was centrifuged and plasma was subsequently frozen at a temperature of −70°C.

### Methods

#### Quantitative polymerase chain reaction (qPCR) analysis

Total RNA was extracted from samples using TRIzol reagent and the RNeasy Mini kit (Qiagen, Hilden, Germany) using the standard procedure (19–21). RNA concentration and purity was determined spectrophotometrically (NanoDrop; ThermoScientific, Waltham, USA). For each sample, 1 μg of total RNA was reversely transcribed using MMLV reverse transcription kit (Novazym, Poznań, Poland) using Oligo dT (PE Biosystems, Warrington, UK) as primers. The reaction was performed at 42.8°C for 60 min (UNO II thermocycler; Biometra, Goettingen, Germany). All primer sets were designed to span, with the exception of CXCR4 variant 1, at least one intron (Table I). The variants studied were those listed in GenBank (*SDF-1*: Gene ID, 6387; *CXCR4:* Gene ID, 7852; *CXCR7*: Gene ID, 57007). Primers were purchased from the Laboratory of DNA Sequencing and Oligonucleotide Synthesis (Institute of Biochemistry and Biophysics, Polish Academy of Sciences, Warsaw, Poland).

qPCR was performed in a Roche Light Cycler 2.0 (Roche, Mannheim, Germany) with software version 4.05. The SYBR Green detection system was used with the above listed primers. qPCR reactions were conducted in 20 μl mixtures, containing 4 μl template cDNA, 0.5 mM of each gene-specific primer and 3.5 mM of Mg^2+^ ions. LightCycler FastStart DNA Master SYBR Green I mix (Roche) was used. The qPCR program included a 10 min denaturation step to activate the Taq DNA polymerase, followed by a three-step amplification program as follows: Denaturation at 95.0°C for 10 sec, annealing at 58.0°C for 5 sec and extension at 72.0°C for 5 sec. Specificity of the reaction products was routinely checked by determination of melting points (0.1°C/sec transition rate) and random sample separation in a 2.0% ethidium bromide/agarose gel. All qPCR reactions were performed in triplicate and the mitochondrial ribosomal protein L19 gene was used as a reference to normalize the data. Templates not submitted to the reverse transcription reaction served as negative controls.

PCR efficiency was assessed by a serial dilution method. Briefly, the products of the qPCR reactions were separated in a 2.0% agarose gel and specific bands were extracted using a DNA gel extraction kit (Millipore, Billerica, MA, USA). The quantity of extracted DNA was estimated spectrophotometrically. Extracted DNA was diluted (10-fold serial dilutions) in order to generate a standard curve for efficiency calculation. The LightCycler software employed (version 4.05) allowed the amplification efficiency to be evaluated from the plots (19–21).

#### Plasma concentrations of SDF-1α and CA 125

Blood samples were obtained from the patients from the antecubital vein between 7 and 8 am following an overnight fast. They were centrifuged at 4°C and subsequently frozen and stored at −70°C.

SDF-1 was quantified using a commercially available EIA kit (Human CXCL12/SDF-1 Immunoassay; R&D Systems Europe Ltd., Abingdon, UK; catalog no. DSA00) according to the manufacturer’s instructions. This kit is specific for the protein encoded by *SDF-1* transcript variant 1, SDF-1α. Absorbance was read at 450 nm (BioTek Synergy 2; BioTek Instruments, Inc., Winooski, VT, USA). All steps were performed at room temperature.

CA 125 was quantified by the automated microparticle enzyme immunosorbent assay method using the AXSYM system of Abbott Laboratories (Chicago, IL, USA).

#### Statistics

The data are expressed as the mean ± SE. Statistical comparison of obtained data was performed by means of the Mann-Whitney U or Wilcoxon tests. All calculations were performed by Statistica 7.0. software (Statsoft, Tulsa, OK, USA).

## Results

### Expression of SDF-1 and CXCR4 transcript variants and CXCR7 in epithelial ovarian cancer and control ovaries

**Figure 1:**
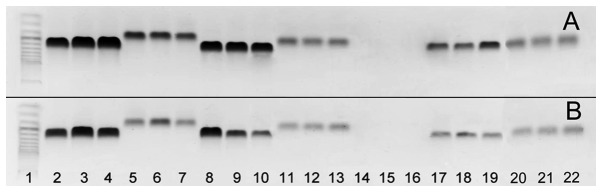
Ethidium bromide-stained 2% agarose gel demonstrating cDNA amplified with specific primer from RNA of (A) three ovarian cancers and (B) three control ovaries. Lane 1, size marker (O’Range Ruler 50-bp DNA Ladder; MBI Fermentas, Lithuania); lanes 2–4, *SDF-1*, transcript variant 1; lanes 5–7, *SDF-1*, transcript variant 2; lanes 8–10, *SDF-1*, transcript variant 3; lanes 11–13, *SDF-1*, transcript variant 4; lanes 14–16, *CXCR4*, transcript variant 1; lanes 17–19, *CXCR4*, transcript variant 2; lanes 20–22, *CXCR7*. In the control and neoplastic ovaries, no reaction product for *CXCR4*, transcript variant 1 was found. For the remaining genes, reaction products with expected size were observed. SDF-1, stromal cell-derived factor-1.

**Figure 2:**
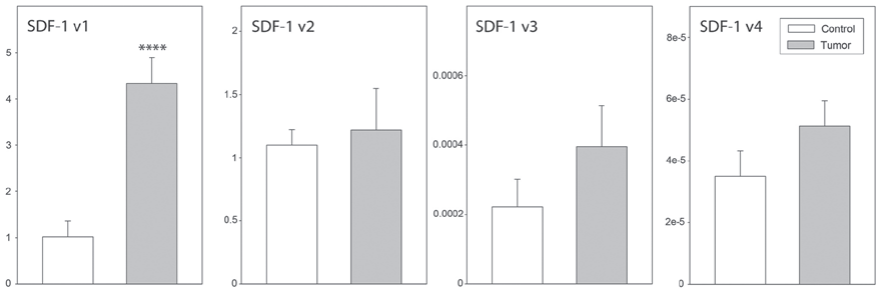
Quantitative polymerase chain reaction assay of *SDF-1*, transcript variants 1–4 mRNA expression in ovarian cancer (n=27) and in control ovaries (n=13). *SDF-1*, transcript variants 1–4 mRNA expression levels were correlated with the expression levels of the mitochondrial ribosomal protein L19 reference gene. Bars represent the mean ± SE. Statistically significant difference in relation to the control group (Mann-Whitney U test): ^****^P<0.001. SDF-1, stromal cell-derived factor-1..

**Figure 3:**
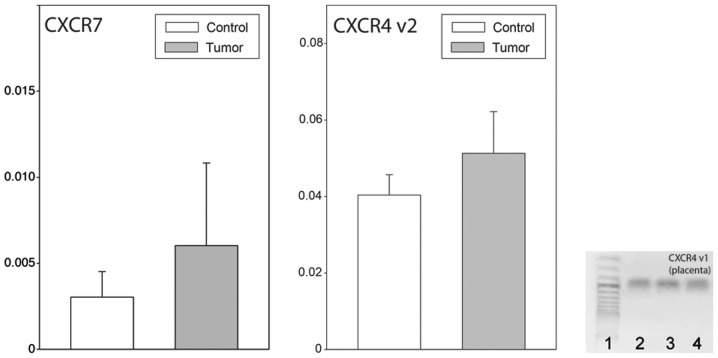
Quantitative polymerase chain reaction assay of *CXCR7* and *CXCR4*, transcript variant 2 mRNA expression in ovarian cancer (n=27) and in control ovaries (n=13). The total number of cases for *CXCR7* was six. Bars represent the relative expression of *CXRX7* and *CXRX4* variant 2 genes in relation to the mitochondrial ribosomal protein L19 reference gene. Results are expressed as the means ± SE. .In the control ovaries and epithelial ovarian cancer, *CXCR4*, transcript variant 1 mRNA expression was not found, while in placenta (lanes 1–3) the signal was well detected indicating proper structure of the primer.

In the epithelial ovarian cancer and control ovary group, agarose gel electrophoresis of classic PCR products revealed expression of *SDF-1*, transcript variants 1–4 (Fig. 1). In all cases, the agarose gel showed bands of the expected size. By contrast, in the control and neoplastic ovaries, no reaction product for *CXCR4* transcript variant 1 was found, while that of variant 2 was highly expressed. It should be emphasized that in human placenta tissue, which was used as a positive control, the reaction products for *CXCR4* transcript variant 1 were abundant (Fig. 2). Furthermore, in normal and neoplastic ovaries, the expression of *CXCR7* was identified. Following this, we studied *SDF-1* transcript variants 1–4 mRNA expression levels by qPCR. As demonstrated in Fig. 3, high *SDF-1* transcript variant 1 expression levels were identified in ovarian cancer and control ovaries. By contrast, in the two groups studied, the expression levels of *SDF-1* transcript variants 3 and 4 were extremely low. Furthermore, the expression levels of *SDF-1* transcript variant 1 were notably higher in epithelial ovarian cancer than in control ovaries (P<0.001), while data for the remaining transcripts were similar in both groups. With regard to *CXCR4* transcript variant 2 and *CXCR7*, their expression levels in normal and neoplastic ovaries were similar (Fig. 3).

### Plasma concentrations of SDF-1α and CA 125 in epithelial ovarian cancer and control patients

**Figure 4:**
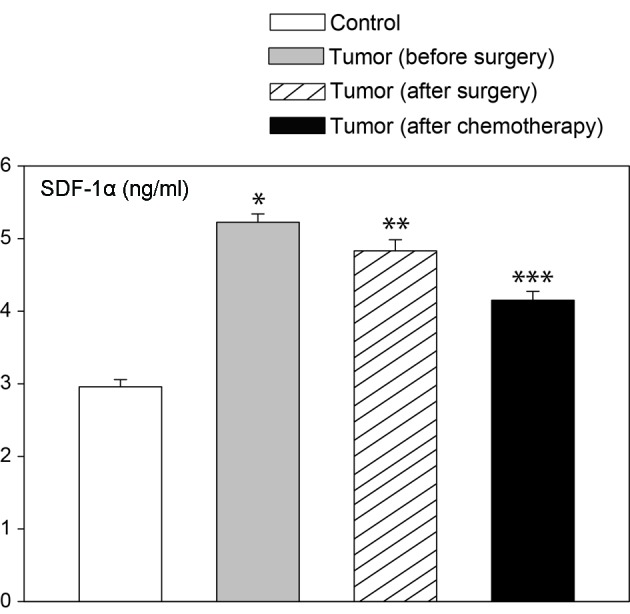
Plasma SDF-1α concentrations (ng/ml) in females with ovarian cancer prior to and after surgery (day 6), and after completion of the first chemotherapy course. The data for 43 cases are shown. The control group includes 30 females. Bars represent the mean ± SE. Statistically significant difference in relation to the control group (Wilcoxon tests): ^*^P<0.05, ^**^P<0.01 and ^***^P<0.001. SDF-1, stromal cell-derived factor-1.

**Figure 5:**
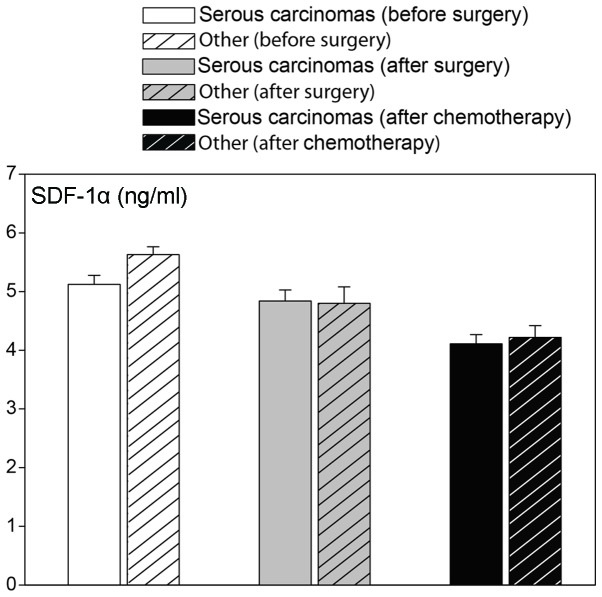
Plasma SDF-1α concentrations (ng/ml) in females with serous and other ovarian cancer prior to and after surgery (day 6), and after completion of the first chemotherapy course. The cases of epithelial ovarian cancer consisted of 29 patients classified as serous and 14 cases classified as other. Bars represent the mean ± SE. SDF-1, stromal cell-derived factor-1.

**Figure 6:**
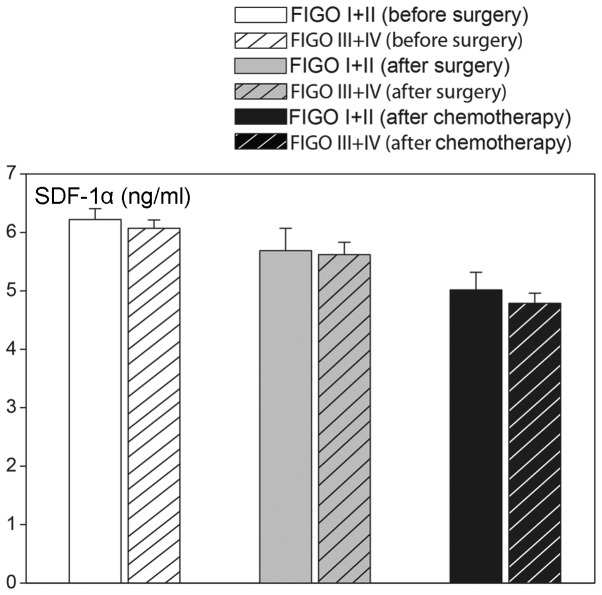
Plasma SDF-1 α concentrations (ng/ml) in females with epithelial ovarian cancer prior to and after (day 6) surgery and after completion of the first chemotherapy course, in relation to FIGO classification. FIGO I + II, n=9; FIGO III + IV, n=34. Bars represent the mean ± SE. SDF-1, stromal-derived factor-1; FIGO, International Federation of Gynecology and Obstetrics.

**Figure 7:**
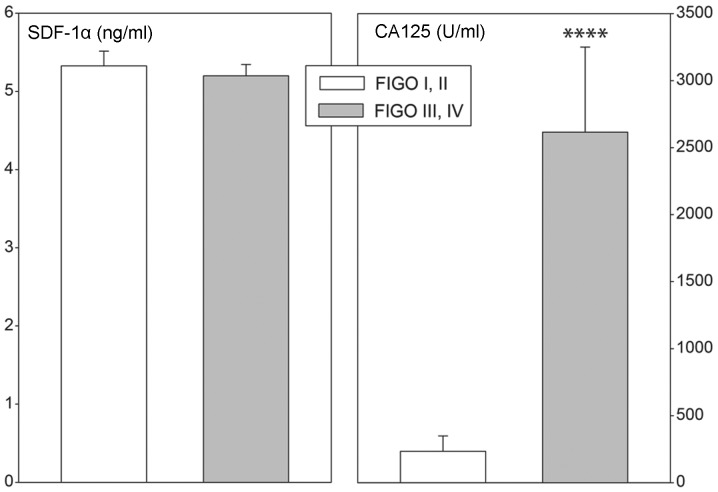
Plasma SDF-1α (ng/ml) and CA 125 (U/ml) concentrations in patients in relation to FIGO classifications for staging ovarian cancer. Open bars represent FIGO I + II (n=9), black bars represent FIGO III + IV (n=34). Bars represent the mean ± SE. Statistically significant difference in relation to FIGO I + II group (Mann-Whitney test): ^****^P<0.001. SDF-1, stromal cell-derived factor-1; CA 125, cancer antigen 125; FIGO, International Federation of Gynecology and Obstetrics.

**Figure 8:**
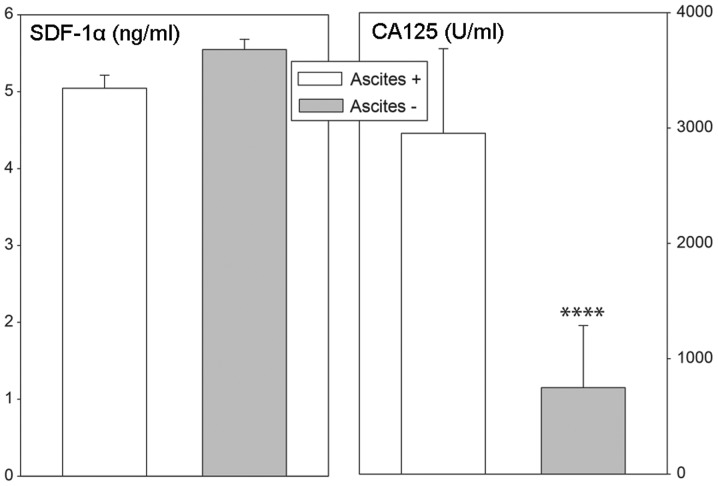
Plasma SDF-1α (ng/ml) and CA 125 (U/ml) concentrations in ovarian cancer patients in relation to ascites. Open bars represent ascites (n=27), black bars represent without ascites (n=34). Bars represent the mean + SE. Statistically significant difference in relation to ascites group (Mann-Whitney test): ^****^P<0.001. SDF-1, stromal cell-derived factor-1; CA 125, cancer antigen 125.

The levels of SDF-1, a circulating chemokine, were quantified in patients using a kit that is specific for SDF-1α, a protein encoded by *SDF-1* transcript variant 1. As demonstrated in Fig. 4, plasma SDF-1α levels were notably higher in females with epithelial ovarian cancer than in control ovaries. Elevated levels of blood SDF-1α were found prior to surgery, 6 days after surgery and following the completion of the first chemotherapy course. These changes were independent of the type of epithelial ovarian cancer, as similar values were observed in serous cancers, as well as other types (Fig. 5). We also analyzed plasma SDF-1α levels in relation to FIGO classifications for ovarian cancer staging. As shown in Fig. 6, elevated levels of SDF-1α were found in less (I and II) and more (III and IV) advanced ovarian cancers (Fig. 7). Plasma CA 125 concentrations were lower in less advanced compared with more advanced cases of cancer. Furthermore, elevated plasma SDF-1α levels in ovarian cancer patients were independent of the presence of ascites, while in cases with ascites, plasma CA 125 concentrations were notably elevated (Fig. 8).

## Discussion

Chemokines are important in the pathogenesis of several tumors. Early studies demonstrated an evident correlation between the expression of chemokine receptors and the prognosis or metastases in various human malignant tumors. This suggests that chemokines and their corresponding receptors are important in controlling key biological properties of the microenvironment in which neoplastic tumors develop (4,15).

SDF-1 is a small cytokine belonging to the CXC chemokine family. Among its multiple functions, SDF-1 is strongly chemotactic for lymphocytes, is able to directly activate leukocytes and can recruit macrophages to malignant tumors. During embryonic development, SDF-1 is important in the migrational behavior of hematopoietic cells, angiogenesis and vasculogenesis. It has also been suggested that this chemokine is involved in cancer metastasis as tumor cells frequently express specific receptors for this chemotactic compound (4,15).

The synthesis of SDF-1 is mainly controlled at the stage of splicing, where alternative splicing events produce a number of different SDF-1 isoforms, all of which are secreted from the cell as functional proteins (22–24). Furthermore, *SDF-1* transcription variants appear to be differentially expressed in various tissues, where they may play different roles.

SDF-1 is synthesized by bone marrow stromal cells and the gene has also been identified to be expressed in numerous other cell types (4,15,24,25). Its expression at the mRNA and protein levels has been demonstrated in tumors, including epithelial ovarian carcinoma. The original data documented SDF-1 expression in >90% of ovarian cancers, in ovarian cancer cell lines and at the protein level, as well as in ascites of ovarian cancer patients. Notably, the expression of SDF-1 was not originally identified in normal ovaries or it was present at extremely low levels (7,8,16,18,26). However, it should be noted, that the expression of SDF-1 in tumors was estimated from immunohistochemsitry, with no attempt made to identify its isoforms or protein levels.

In this study, using qPCR, we have identified the expression of four *SDF-1* transcriptional variants in epithelial ovarian cancer. There are numerous human SDF-1 isoforms (α-ϕ) (24), the present study was based on current data included in GenBank (*SDF-1*: Gene ID, 6387; *CXCR4*: Gene ID, 7852; *CXCR7*: Gene ID, 57007). The results of our studies have demonstrated that in the control and neoplastically altered ovaries, the highest expression level was exhibited by *SDF-1* variant 1, a lower expression by variant 2 and trace expression by variants 3 and 4. Such a pattern of transcript expression corroborates the earlier observation that *SDF-1* variant 1 is the most widespread splicing variant (27). It is known that *SDF-1* is constitutively expressed in tissues (10,28). While the expression levels of other variants were not altered, a marked elevation in the expression of *SDF-1* transcript variants in epithelial ovarian cancer was observed. The results may suggest that in epithelial ovarian cancer, the expression of *SDF-1* and of genes controlling alternative splicing become elevated, leading to increased formation of *SDF-1* variant 1. As revealed in earlier immunocytochemical studies on epithelial ovarian cancer, the expression of SDF-1 mainly takes place in epithelial cells (16,18,26). While our studies have not permitted us to identify specifically which cells demonstrate enhanced expression of *SDF-1* variant 1 in epithelial ovarian cancer types, it cannot be excluded that the expression may be linked to cells recruited for tumor formation. The mechanism(s) that can predict the upregulation of *SDF-1* variant 1 expression in epithelial ovarian cancer is not yet recognized and remains to be elucidated. However, it should be mentioned that earlier studies have implicated tissue hypoxia (which may be present in epithelial ovarian tumors) as a potent factor in upregulating the expression of *SDF-1* and *CXCR4* in various cells and organs (4,28).

CXCR4 is a cognate receptor of SDF-1 (29–31). SDF-1-induced CXCR4 activation results in the influx of calcium ions, as well as the activation of other intracellular signaling pathways, for example MAPK, p42/44-ELK-1, PI3K-AKT-NF-κB and JAK2 and JAC3 (4,15,32). The SDF-1/CXCR4-CXCR7 system plays a pivotal role in organogenesis, regeneration and tumorigenesis. Experimental data indicate that CXCR4-expressing cells follow an SDF-1 gradient (15,32–34). It is frequently emphasized that CXCR4 is the most widely expressed chemokine receptor in malignancy. Numerous, if not all neoplastic cells express this receptor and, therefore, the SDF-1/CXCR4-CXCR7 system may be of particular importance in tumor metastasis (7,8,32). Prevailing data on CXCR4 expression in several types of cancer were obtained by immunohistochemistry; however, the commonly used anti-CXCR4 antibody is only able to recognize a subpopulation of CXCR4 molecules (15–18).

Limited data are available with regard to CXCR4 mRNA expression in epithelial ovarian cancer. Elevated CXCR4 mRNA expression has recently been reported in ovarian cancer; however, this particular study lacked important information regarding the oligonucleotide sequences applied and failed to include measurements of the resultant CXCR4 protein expression (35). Furthermore, the authors did not estimate the known *CXCR4* transcription variants. In this regard, our study has demonstrated that in the control and neoplastic ovaries, *CXCR4* transcript variant 2 was highly expressed while its transcript variant 1 was absent. Furthermore, the expression levels of *CXCR4* transcript variant 2 in normal ovaries and epithelial ovarian cancer were similar. The latter finding was unexpected and the explanation for this result remains to be elucidated. In scope of numerous immunohistochemistry derived data, it seems legitimate to suggest that in normal ovaries and epithelial ovarian cancer, *CXCR4* transcript variant 2 may be translated into a functional protein.

For a number of years, CXCR4 was considered to be the only receptor binding to SDF-1 and it was not until 2005 that CXCR7 was identified as another SDF-1-binding receptor (3,5,36). Experimental data suggest that due to its expression profile within tumor cells, CXCR7 may also be important in tumor growth and metastasis, but investigations of the role of this receptor system in ovarian cancer have been lacking until now. As demonstrated in the present study, the expression levels of *CXCR7* in normal and neoplastic ovaries are similar. Thus, we demonstrated that in epithelial ovarian cancer, CXCR4 and CXCR7 mRNA levels remained unchanged.

Experimental data suggest that all proteins encoded by particular *SDF-1* transcription variants are secretory proteins (24). Of these transcripts, *SDF-1* transcript variant 1 encodes circulating chemokine SDF-1α, which is the main SDF isoform in the blood. Plasma levels of this cytokine were found to be elevated in breast cancer (37), pelvic inflammatory disease (38) and in various systemic diseases (39–41). In ovarian cancer patients, using human cytokine microarray technology, plasma SDF-1 levels were found to be elevated 6.6-fold compared with the control group (42). We have extended these earlier findings by demonstrating in the present study notably higher plasma SDF-1α levels in females with epithelial ovarian cancer. Elevated plasma SDF-1α levels were independent of the type of epithelial ovarian cancer or the stage of the cancer. In addition, these levels remained unaffected by surgery or by subsequent chemotherapy, and were similar in patients with and without ascites. These results indicate no direct correlation between epithelial ovarian cancer and plasma SDF-1α levels. With regard to these observations, it seems legitimate to suggest that elevated plasma SDF-1α levels in epithelial ovarian cancer patients are not correlated to the presence of tumor and/or metastases; however, they rather reflect a general response to the disease. These findings are in contrast to data obtained in breast cancer patients, where plasma SDF-1α levels had a significant correlation with tumor grade and epithelial subtype (37). Thus, in our opinion, plasma SDF-1α levels cannot be used as a marker of epithelial ovarian cancer advancement or progression. Furthermore, we would like to highlight the fact that, in epithelial ovarian cancer, plasma SDF-1α levels demonstrate no correlation with blood CA 125 levels.

The available literature contains numerous studies on the prognostic value of the SDF-1/CXCR4-CXCR7 system in numerous tumor types, including epithelial ovarian cancer. In this regard, the majority of data derives from immunohistochemical studies (including semiquantitative and microarray techniques) and they are frequently correlated with comprehensive databases of clinicopathological variables (18,43). In general, these studies indicate that a high level of SDF-1 expression in ovarian cancer is an independent prognostic factor for tumor progression and a predictor of poor survival (16,43,44).

## Figures and Tables

**Table I:** Conventional qPCR analyses of *CXCL12*, transcript variants 1–4; *CXCR4*, transcript variants 1–2 and *CXCR7*.

cDNA	GenBank accession number	Primer	Primer sequence (5′-3′)	Position	PCR product size (bp)
CXCL12, transcript variant 1	NM_199168	S	TACAGATGCCCATGCCGATT	174–193	262
		A	GCCCTTTCATCTCTCACAAGGT	414–435	
CXCL12, transcript variant 2	NM_000609	S	TGTGCATTGACCCGAAGCTA	301–320	144
		A	CAGGCCCTTCCCTAACACT	426–444	
CXCL12, transcript variant 3	NM_001033886	S	ACTGTGCCCTTCAGATTGTAGCC	253–275	260
		A	AGCAAATTTACAAAGCGCCGAGA	490–512	
CXCL12, transcript variant 4	NM_001178134	S	TACAGATGCCCATGCCGATT	174–193	196
		A	CGCTGATCAGGTTGTTTAAAG	349–369	
CXCR4, transcript variant 1	NM_001008540	S	CTACATTAATTCTCTTGTGCC	175–195	241
		A	ATTTTCTTCACGGAAACAGG	396–415	
CXCR4, transcript variant 2	NM_003467	S	CTGAGTGCTCCAGTAGCC	61–68	281
		A	TGCAGCCTGTACTTGTCC	314–331	
CXCR7	NM_020311.2	S	CAAAGCTGCCATCTAGAGG	16–34	252
		A	CTGATGTCCGAGAAGTTCC	249–267	
MRLP19 (reference gene)	NM_014763	S	TCCTCGGGTCCAGGAGATT	529–547	58
		A	CAAGCTATCATCCAGCCGTTT	566–586	

Oligonucleotide sequences for S and A primers. *MRPL19* was the reference gene. S, sense; A, antisense; MRPL19, mitochondrial ribosomal protein L19; qPCR; quantitative polymerase chain reaction.
